# Factors impacting antibody kinetics, including fever and vaccination intervals, in SARS-CoV-2-naïve adults receiving the first four mRNA COVID-19 vaccine doses

**DOI:** 10.1038/s41598-024-57931-0

**Published:** 2024-03-27

**Authors:** Tomoka Matsuura, Wakaba Fukushima, Yu Nakagama, Yasutoshi Kido, Tetsuo Kase, Kyoko Kondo, Natsuko Kaku, Kazuhiro Matsumoto, Asae Suita, Emiko Mukai, Yuko Nitahara, Ayako Konishi, Ayane Kasamatsu, Sachie Nakagama, Etsuko Nakagami-Yamaguchi, Satoko Ohfuji, Yukihiro Kaneko, Akira Kaneko, Hiroshi Kakeya, Yoshio Hirota

**Affiliations:** 1 Department of Public Health, Graduate School of Medicine, Osaka Metropolitan University, 1-4-3, Asahi-Machi, Abeno-ku, Osaka, 545-8585 Japan; 2 Research Center for Infectious Disease Sciences, Graduate School of Medicine, Osaka Metropolitan University, Osaka, Japan; 3 Osaka International Research Center for Infectious Diseases, Osaka, Japan; 4 Department of Virology and Parasitology, Graduate School of Medicine, Osaka Metropolitan University, Osaka, Japan; 5 Management Bureau, Osaka Metropolitan University Hospital, Osaka, Japan; 6 Department of Medical Quality and Safety Science, Graduate School of Medicine, Osaka Metropolitan University, Osaka, Japan; 7 Department of Bacteriology, Graduate School of Medicine, Osaka Metropolitan University, Osaka, Japan; 8 Department of Infection Control Science, Graduate School of Medicine, Osaka Metropolitan University, Osaka, Japan; 9 Clinical Epidemiology Research Center, SOUSEIKAI Medical Group (Medical Co. LTA), Fukuoka, Japan

**Keywords:** Risk factors, Epidemiology

## Abstract

To evaluate the antibody response following the initial four doses of mRNA vaccines (BNT162b2 or mRNA-1273) in SARS-CoV-2-naïve healthy adults and investigate factors influencing antibody titer increases, this prospective cohort study was conducted in Japan from March 2021. The study included participants who received either the 1st and 2nd doses (n = 467), 3rd dose (n = 157), or 4th dose (n = 89). Blood samples were collected before and up to 6 months after each dose, and anti-receptor-binding domain antibody levels were measured. Multivariate analysis (usin multiple linear regression or linear mixed models) revealed several factors significantly associated with higher post-vaccination antibody levels, including mRNA-1273 vaccine (after the 1st and 2nd dose), male gender (after the 3rd and 4th doses), younger age (after the 1st and 2nd dose), non-smoking status (after the 2nd dose), non-use of immunosuppressive agents (after the 1st dose), higher pre-vaccination antibody titers (after the 2nd, 3rd, and 4th doses), and higher post-vaccination fever (after the 2nd and 4th doses). Furthermore, longer intervals since the last dose were significantly associated with higher antibody levels after the 3rd and 4th doses. These findings provide valuable insights for optimizing vaccination strategies.

## Introduction

The pandemic of the coronavirus disease 2019 (COVID-19) spread worldwide and had unprecedented impacts. However, with the acquisition of herd immunity, the pandemic gradually subsides by 2023. One significant contributor to achieving herd immunity was the global administration of vaccines such as Pfizer-BioNTech's BNT162b2 and Moderna's mRNA-1273 developed against severe acute respiratory syndrome coronavirus 2 (SARS-CoV-2)^1–3^. The immunogenicity of these vaccines can be evaluated by measuring the levels of antibodies against the receptor-binding domain (RBD). It has been demonstrated that anti-RBD antibody titers strongly correlate with virus neutralizing activity and vaccine efficacy^4–6^. However, there have been limited studies investigating the kinetics of antibody titers from the first to the fourth dose in a relatively large cohort.

Considering individual variations in immune response to vaccination, several studies have explored factors influencing immune response to COVID-19 mRNA vaccines. Factors reported to influence the immune response to the primary vaccination series (first and second doses) include prior infection, age, gender, obesity, use of immunosuppressive agents, smoking, alcohol drinking, comorbidities such as hypertension, post-vaccination adverse reactions, and the interval between the first and second doses^7–15^. However, there is limited research on factors influencing antibody titers after the third^15–17^ and fourth^18,19^ doses, especially regarding the dosing interval from the last vaccination.

In our previous article^7^, we reported interim results on the kinetics of anti-RBD antibody titers for six months after the primary series (first and second doses) of vaccination, as well as the impact of age, sex, and body mass index (BMI) on post-vaccination antibody titers in a cohort predominantly consisting of Japanese healthcare workers. Extending the observation period of the previous article and adding information on subsequent vaccinations, the present study aimed to investigate the kinetics of anti-RBD antibody levels after the first, second, third, and fourth doses of mRNA COVID-19 vaccine in healthy adults without a history of SARS-CoV-2 infection. Furthermore, due to an increased number of participants receiving the primary doses compared to our previous report^7^, this study additionally examined the influence of factors such as vaccine product, smoking, alcohol drinking, use of immunosuppressive agents, hypertension, diabetes, dyslipidemia, pre-vaccination antibody titers, post-vaccination fever level, and intervals between each dose on the kinetics of antibody levels after each vaccination.

## Method

### Study design

We combined data from two prospective cohort studies which were conducted at Osaka Metropolitan University Hospital in Japan. Information on participants who received the first and second doses of vaccine was obtained from the first study conducted from March 2021 to June 2022. The eligibility criteria for the first study were as follows: (1) aged between 20 and 64 years at registration, (2) a healthcare worker at Osaka Metropolitan University Hospital, an employee of the Osaka City Health Bureau, a faculty member or student at the medical school or nursing school of Osaka Metropolitan University, or a clinical trial volunteer at Osaka Metropolitan University Hospital, and (3) an individual who voluntarily provided written consent to participate in the study. Those with a history of COVID-19 infection or COVID-19 vaccination or with contraindications to vaccination were excluded.

Information on participants who received the third and fourth doses of vaccine was obtained from the second study being conducted at the same hospital from February 2022 followed by the first study. This report includes information obtained up to March 2023. The eligibility criteria were those who participated in the first study or those who met as follows: (1) those who were ≥ 20 years old, (2) those who had received their second COVID-19 vaccine ≥ 5 months prior, (3) a healthcare worker at Osaka Metropolitan University Hospital, or a faculty member or student of the medical school at Osaka Metropolitan University, and (4) an individual who voluntarily provided written consent to participate in the study. The two study protocols were developed in compliance with the Helsinki Declaration and approved by the Osaka Metropolitan University Hospital Certified Review Board (approval numbers: OCU010E, OCU013E, registration numbers: jRCT1051200143, jRCT1051210161). After the nature of the study and the potential outcomes were sufficiently explained, written informed consent was obtained from the participants.

### Vaccination, samples, and data collection

Information regarding the participants' basic characteristics, such as occupation, gender, age, height, weight, systemic use of immunosuppressive agents within 6 months, underlying diseases, smoking status, drinking status and details of adverse reactions following COVID-19 vaccination, including the highest body temperature at 0.5 °C intervals within 48 h after vaccination, were self-reported by the participants using a study-specific electronic data capture system.

All participants received monovalent mRNA vaccines targeting the wild-type SARS-CoV-2 as specified in the package insert. In Japan, individuals were able to choose which vaccine to receive. However, in practice, the type of vaccine (BNT162b2 or mRNA-1273) was determined based on the location and timing of vaccination. For BNT162b2, the standard interval between the first and second doses was 21 days. Participants received a 0.3 mL diluted BNT162b2 vaccine per dose. For mRNA-1273, the standard interval between the first and second doses was 28 days. In the first and second vaccinations, a 0.5 mL dose of the vaccine was administered, while in the third and fourth vaccinations, a 0.25 mL dose was administered. All doses were provided via intramuscular injection in the deltoid muscle.

Blood samples were collected at pre-determined time points: within 1 week before the first dose (V1-0), within 1 week before the second dose (V2-0), 4–5 weeks after the second dose (V2-4W), and 6 months after the second dose (V2-6M). For the third dose, blood samples were collected within 2 weeks before the dose (V3-0), 7–17 days after the dose (V3-2W), 3 months after the dose (V3-3M), and 6 months after the dose (V3-6M). Similarly, for the fourth dose, blood samples were collected within 2 weeks before the dose (V4-0), 7–17 days after the dose (V4-2W), 3 months after the dose (V4-3M), and 6 months after the dose (V4-6M).

### Measurement of antibody titers

Anti-RBD antibody titers were measured using Architect SARS-CoV-2 IgG II Quant (Abbott Laboratories, Illinois, USA), with a quantitative range of 6.8 to 120,000 (AU/mL) in this study, and the cut-off value for anti-RBD test positivity was 50 (AU/mL) or higher^20^. Anti-SARS-CoV-2 nucleocapsid protein (anti-N) titers were measured using the Elecsys anti-SARS-CoV-2 (Roche Diagnostics, Basel, Switzerland) with a positive anti-N test cutoff of ≥ 1.0, which was considered to have a history of infection^21^. We selected specific assays for detecting anti-RBD and anti-N antibodies based on their characteristics. The Architect assay was chosen for anti-RBD detection due to its wider dynamic range and correlation with viral neutralizing capacity^22^. On the other hand, the Elecsys assay demonstrated superior long-term detection capability for prior infection^23,24^.

### Selection of participants for analysis

Participants included in the analysis of the first and second vaccinations were those who participated in the first study, received the first and second dose, had four blood samples (V1-0, V2-0, V2-4W, V2-6M) collected on schedule, provided information on adverse reactions, and showed no increase in anti-N antibodies at the time of blood sampling 6 months after the second dose (V2-6M). Participants included in the analysis of the third vaccination were those who participated in the second study, received the third dose, had four blood samples (V3-0, V3-2W, V3-3M, V3-6M) collected on schedule, provided information on adverse reactions, and showed no increase in anti-N antibodies at the time of blood sampling 6 months after the third dose (V3-6M). The participants included in the analysis of the fourth dose of the vaccine were those who participated in the second study, received the fourth dose of the vaccine, and completed on schedule with at least the first three blood samples (V4-0, V4-2W, V4-3M) without an increase in anti-N antibodies. The blood samples at V4-6M were only used in the analysis if they were taken and did not show an increase in anti-N antibody titers.

### Statistical analysis

BMI (kg/m^2^) was calculated as weight/(height)^2^ and classified into three categories based on the Japanese criteria for underweight (< 18.5), normal weight (18.5–24.9), or obesity (≥ 25)^25^. Current smoking and drinking statuses were classified into two categories (no and yes). The level of post-vaccination fever was divided into three categories: < 37 °C, 37.0–37.9 °C, and ≥ 38 °C. The pre-vaccination anti-RBD titers and the interval between the doses were classified into three categories based on < 25th percentile, 25th-75th percentile, and > 75th percentile of their respective distributions, as approximately half of the participants were vaccinated at intervals around the median.

The geometric mean anti-RBD antibody titers (GMTs) were calculated at each time point, serving as an average of the logarithms for a set of antibody measurements. Additionally, the geometric mean ratios between pre-vaccination and post-vaccination (GMT ratios) were calculated, showing the increase in antibody titers due to each vaccination. Participants were stratified by vaccine product (BNT162b2 or mRNA-1273), gender, age group (20–39 years, 40–49 years, and 50 years and older), BMI category, smoking status, drinking status, use of immunosuppressive agents, hypertension, diabetes, dyslipidemia, pre-vaccination antibody titer category, the level of post-vaccination fever category, and the interval between the doses categories. The GMT and GMT ratio between categories were compared using the Wilcoxon rank-sum test or Jonckheere-Terpstra trend test. Multivariate analysis examined factors that predict base 10 log-transformed antibody titers after each dose. For predictor of the antibody titers after the first vaccination (V2-0), a multiple linear regression model was used, while a linear mixed-effects model was used for predictor of the antibody levels after the second vaccination (V2-4W, V2-6M), third vaccination (V3-2W, V3-3M, V3-6M), and fourth vaccination (V4-2W, V4-3M, and, if available, V4-6M). All models included vaccine product, gender, age (continuous), BMI (continuous), use of immunosuppressive agents, underlying diseases such as hypertension, diabetes, dyslipidemia, smoking status, alcohol drinking status, log10-transformed pre-vaccination antibody titers, and post-vaccination fever level category as explanatory variables. For the models of the third and fourth vaccinations, the interval since the last vaccinations was added as an explanatory variable. All the models included the number of days from vaccination to each blood sampling (continuous) as an adjustment variable. For the linear mixed-effects models, the participant ID numbers are included as a random-effects variable.

## Results

### Participants

A flow diagram showing the number of participants included in the analysis for each dose recipient is presented in Supplementary Fig. 1. Participant characteristics are shown in Table 1.

**Table 1:** Patient characteristic.

Values in the table represent n (%) unless otherwise indicated		The first and second dose recipients (N = 467)	The third dose recipients (N = 157)	The fourth dose recipients (N = 89)	
Occupation	Healthcare worker	293 (63)	56 (36)	47 (53)	
	Others	174 (37)	101 (64)	42 (47)	
Administered vaccine product	BNT162b2	454 (97)	149 (95)	34 (38)	
	mRNA-1273	13 (3)	8 (5)	55 (62)	
Gender	Male	145 (31)	17 (11)	17 (19)	
	Female	322 (69)	140 (89)	72 (81)	
Age group	(Median, IQR)	43, 33.5–50.5	46, 38–52	49, 42–55	
	20–39	177 (38)	42 (27)	20 (23)	
	40–49	156 (33)	62 (40)	29 (33)	
	50-	134 (29)	53 (34)	40 (45)	
Body mass index	(Median, IQR)	21.5, 19.7–23.6	21.2, 19.3–23.0	21.3, 19.9–23.0	
	< 18.5	61 (13)	27 (17)	6 (7)	
	18.5–24.9	328 (70)	115 (73)	69 (78)	
	≥ 25	78 (17)	15 (10)	14 (16)	
Smoking status	Yes	18 (4)	10 (6)	5 (6)	
Drinking status	Yes	282 (60)	73 (47)	43 (48)	
Use of immunosuppressive agents	Yes	9 (2)	0 (0)	1 (1)	
Underlying diseases	Hypertension	47 (10)	10 (6)	10 (11)	
	Dyslipidemia	41 (9)	11 (7)	10 (11)	
	Diabetes	9 (2)	2 (1)	1 (1)	

		After the first dose	After the second dose	After the third dose	After the fourth dose
The level of post-vaccination fever	< 37 °C	423 (91)	191 (41)	61 (39)	36 (40)
	37.0–37.9 °C	41 (9)	195 (42)	71 (45)	35 (39)
	≥ 38 °C	3 (1)	81 (17)	25 (16)	18 (20)

		The first and second doses	The second and third doses	The third and fourth doses	
The interval between the doses (days)	Median, IQR	21, 21–21	266, 260–269	235, 207–244	
	< 25th percentile	4 (1)	32 (20)	22 (35)	
	25th–75th percentile	436 (93)	88 (56)	45 (51)	
	> 75th percentile	27 (6)	37 (24)	22 (25)	
Vaccine products administered in the first and second doses	BNT162b2		153 (98)	89 (100)	
	mRNA-1273		4 (3)	0 (0)	
Vaccine products administered in the third dose	BNT162b2			88 (99)	
	mRNA-1273			1 (1)	

A total of 467 participants were included in the analysis of the first and second dose. Approximately 63% of the participants were healthcare workers. Most participants (97%) received the BNT162b2 vaccine. About two-thirds of the participants were female, the median age was 43 years, and 70% had a normal BMI. Current smokers accounted for 4% of the participants, while 60% were current drinkers. Only 2% of participants reported systemic use of immunosuppressive agents. Ten percent had hypertension, 2% had diabetes, and 9% had dyslipidemia. Fever levels after the first and second doses were below 37 °C for 91% and 41% of the participants, respectively. Fever above 38 °C were observed in 1% after the first dose and 17% after the second dose. The majority (93%) had a 21-day interval between the first and second vaccinations. For the analysis of the third dose, 157 participants were included. Females accounted for 89% of the recipients, and the median age was 46 years. The median interval from the second dose to the third dose was 266 days. For the analysis of the fourth dose, 89 participants were included, but only 33 of them had blood drawn 6 months after vaccination (V4-6M) and tested negative for N antibodies. More than half (62%) received the mRNA-1273 vaccine. The majority (81%) were females, and median age was 49 years. The median interval from the third dose to the fourth dose was 235 days.

### Kinetics of antibody titers over four doses

**Figure 1:**
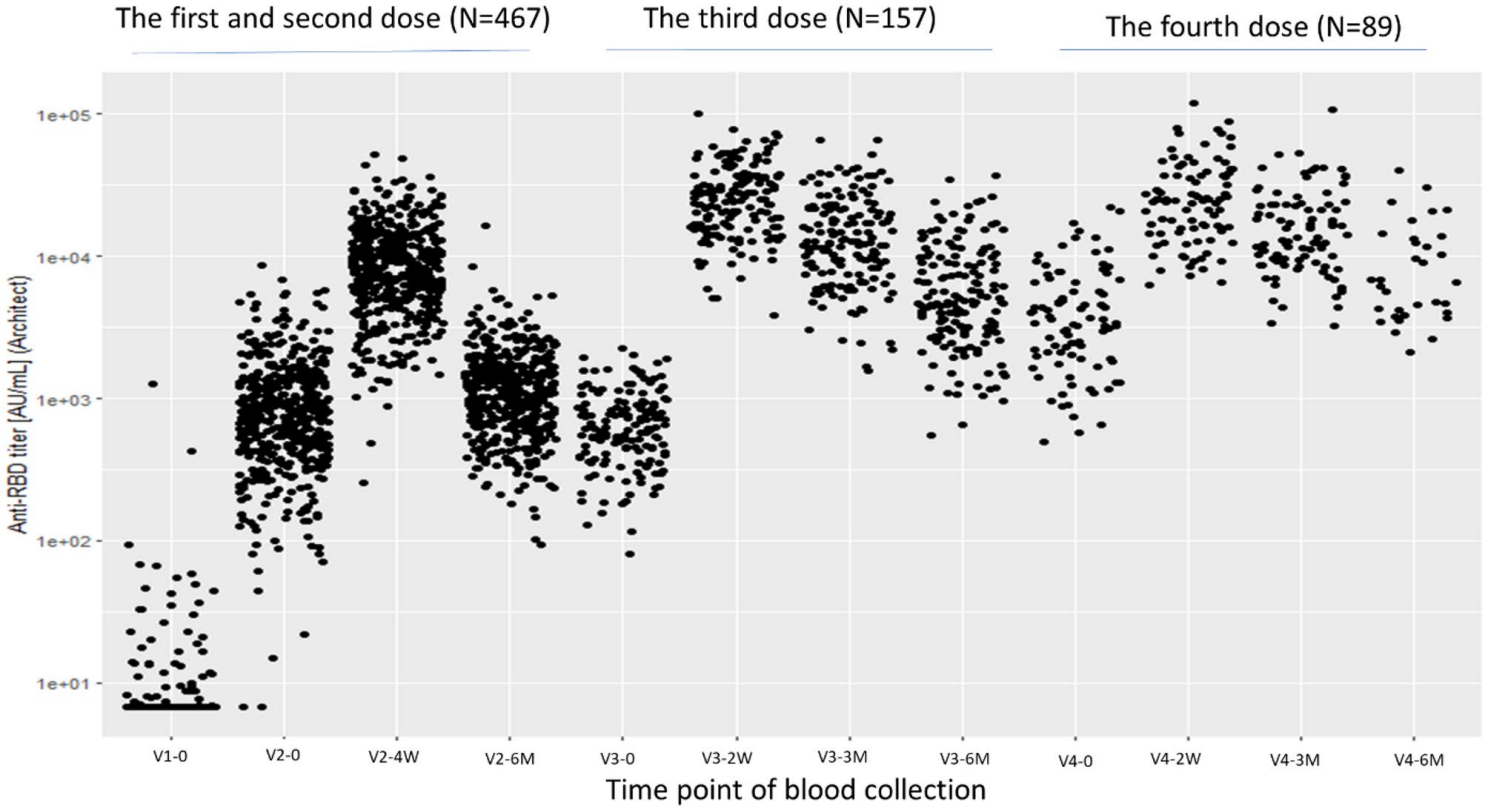
Kinetics of antibody titers over four doses. Time point of blood sampling are as follows: V1-0: within 1 week before the first dose, V2-0: within 1 week before the second dose, V2-4W: 4–5 weeks after the second dose, V2-6M: 6 months after the second dose, V3-0: within 2 weeks before the third dose, V3-2W: 7–17 days after the third dose, V3-3M: 3 months after the third dose, V3-6M: 6 months after the third dose, V4-0: within 2 weeks before the fourth dose, V4-2W: 7–17 days after the fourth dose, V4-3M: 3 months after the fourth dose, V4-6M: 6 months after the fourth dose.

**Figure 2:**
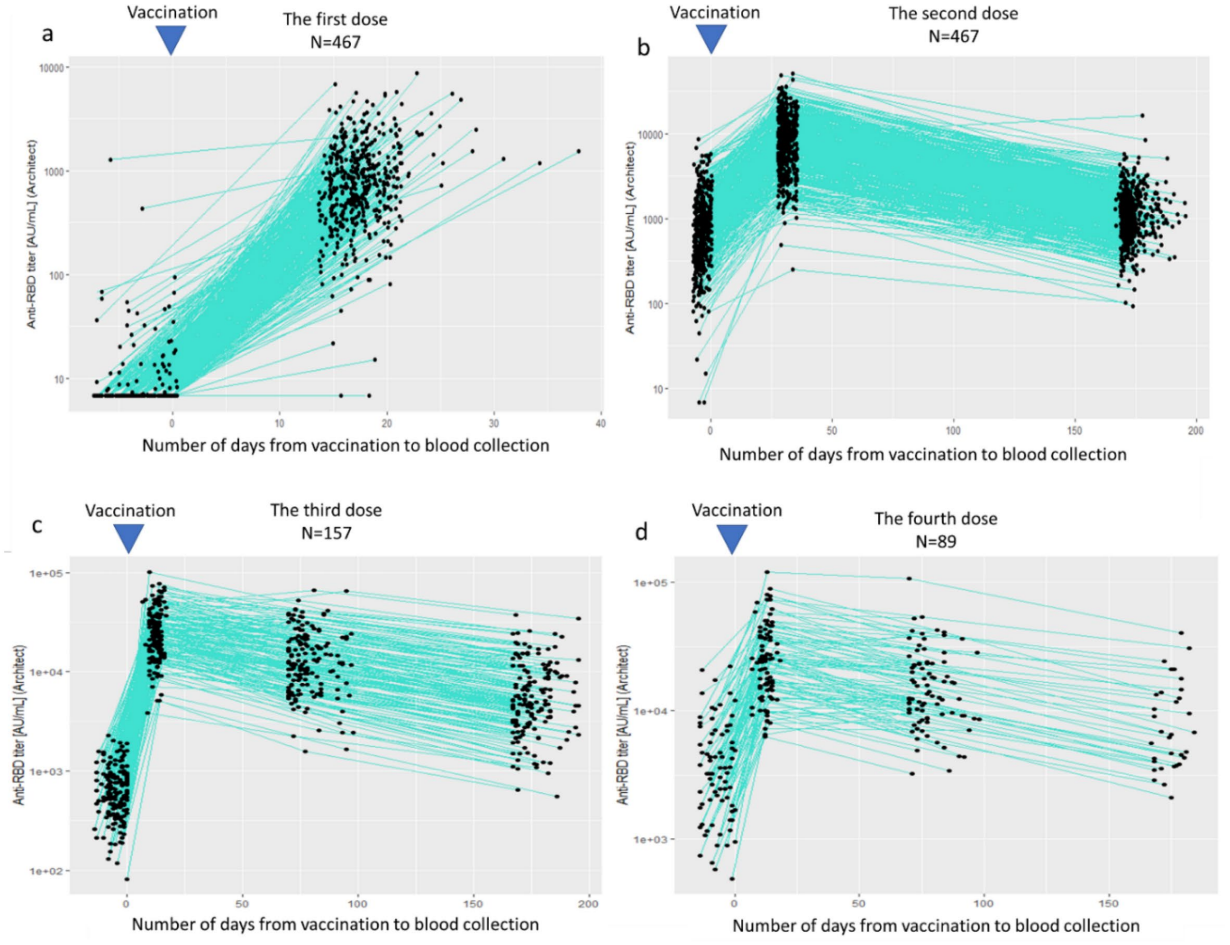
Kinetics of antibody titers before and after the first, second, third, and fourth doses, respectively.

Figure 1 shows a scatter plot of anti-RBD antibody titers at each blood sampling time point from V1-0 to V4-6M. Figure 2a–d show the kinetics of antibody titers before and after the 1st, 2nd, 3rd, and 4th doses, respectively. After the first dose, there was variability in the increase of antibody titers between individuals. However, antibody titers following the second, third, and fourth doses showed less variability, with substantial and rapid increases in all participants, followed by gradually declining over a period of 6 months.

Table 2 shows the overall GMTs and GMT ratios for each blood collection time point. The GMTs 6 months after each vaccination were higher than the pre-vaccination titers. When comparing the GMTs of antibody titers 6 months after the second, third, and fourth vaccinations, the highest GMT was observed after the fourth vaccination (V4-6M: 7473 AU/mL). Conversely, the GMT ratio in antibody titer 6 months after vaccination was highest after the third dose (V3-6M/V3-0: 8.7). The pre-vaccination GMTs prior to the second and third doses were similar (V2-0: 727 AU/mL vs. V3-0: 601 AU/mL). In contrast, both the GMT and GMT ratio 6 months after the third vaccination markedly exceeded those of the second vaccination (V2-6M: 1078 AU/mL vs. V3-6M: 5199 AU/mL; V2-6M/V2-0: 1.5 vs. V3-6M/V3-0: 8.7). For the fourth dose, while the pre-vaccination antibody titer (GMT [AU/mL]) substantially exceeded that of the third dose (601 vs. 3445), the GMTs at 2 weeks (24,224 vs. 23,807), 3 months (12,413 vs. 14,697), and 6 months post-vaccination (5199 vs. 7473) were close.

**Table 2:** Overall geometric mean titers (AU/mL) and geometric mean fold increases (GMT ratios).

	The first and second dose (N = 467)				The third dose (N = 157)				The fourth dose (N = 89)			
	V1-0	V2-0	V2-4W	V2-6M	V3-0	V3-2W	V3-3M	V3-6M	V4-0	V4-2W	V4-3M	V4-6Ma
Geometric mean titer	7.7	727	7804	1078	601	24,224	12,413	5199	3445	23,807	14,697	7473
25th percentile	6.8	429	5087	700	408	16,263	7467	3073	2008	14,968	9193	4050
Median	6.8	763	8361	1123	618	25,118	12,302	4890	3397	24,529	13,512	6505
75th percentile	6.8	1333	13,141	1662	915.7	36,626	21,432	9798	6564	37,310	23,194	13,424

	The first dose	The second dose		The third dose			The fourth dose					
	V2-0/V1-0	V2-4W/V2-0	V2-6M/V2-0	V3-2W/V3-0	V3-3M/V3-0	V3-6M/V3-0	V4-2W/V4-0	V4-3M/V4-0	V4-6M^a^/V4-0			
Geometric mean fold increase	94.8	10.7	1.5	40.3	20.7	8.7	6.9	4.3	2.1			
25th percentile	57.7	6.6	0.9	28.0	13.5	5.5	4.4	2.8	1.3			
Median	104.5	10.1	1.4	41.4	20.2	8.5	7.6	4.0	1.9			
75th percentile	188.9	16.8	2.2	54.8	30.3	13.6	10.8	5.6	2.8			

^a^Antibody titers at V4-6M were missing in 56 cases.

### GMTs and GMT ratios of anti-RBD antibodies: comparison between each category

The GMTs were examined based on subjects' characteristics (Table 3). Significant differences were observed based on vaccine products (V2-0, V2-4W, V2-6M, V3-0, V3-2W), gender (V3-3M, V3-6M), age group (V2-0, V2-4W, V2-6M), BMI category (V2-6M, V4-2W), smoking status (V2-6M, V3-0), use of immunosuppressive agents (V3-0), hypertension (V2-4W, V2-6M, V3-0, V4-0), and dyslipidemia (V2-6M). No significant differences based on drinking status were observed at any time point. Significant differences were evident at all time points based on pre-vaccination antibody titer. There were numerous points after each vaccination with significant differences based on the level of post-vaccination fever (V2-0, V2-4W, V2-6M, V3-2W, V4-2W, V4-3M, V4-6M). Additionally, the pre-vaccination GMTs before the first, second, and fourth doses (V1-0, V2-0, and V4-0) significantly differed based on the fever levels after each dose. As a supplementary analysis, the association between antibody titer categories before each dose and fever levels after each dose was tested using the Chi-square test or Fisher's exact test (Supplementary Table 1). Among the first to fourth vaccinations, a significant association was observed between the antibody titer before the second vaccination and the fever level after the second vaccination (P < 0.01). Furthermore, when stratified by the interval since the last vaccination, a significant difference in antibody titers at V4-3M was observed.

**Table 3:** Geometric mean titers of anti-RBD antibodies (AU/mL) and comparison between each category.

		The first and second dose (N = 467)				The third dose (N = 157)				The fourth dose (N = 89)				
		V1-0	V2-0	V2-4W	V2-6M	V3-0	V3-2W	V3-3M	V3-6M	V4-0	V4-2W	V4-3M	V4-6M^a^	
Vaccine product	BNT162b2	7.6	700	7613	1052	578	23,525	12,180	5089	3407	22,978	12,741	8655	
	mRNA-1273	8.7	2634	18,599	2494	1236	41,792	17,659	7740	3469	24,335	16,054	7073	
	P-value	0.59	** < 0.01**	** < 0.01**	** < 0.01**	** < 0.01**	**0.01**	0.14	0.13	0.79	0.52	0.09	0.52	
Gender	Male	7.8	731	7133	1077	611	30,634	18,696	8101	4118	30,471	17,380	8703	
	Female	7.6	725	8127	1078	599	23,543	11,810	4927	3303	22,460	14,127	7272	
	P-value	0.12	0.60	0.17	0.96	0.83	0.11	**0.02**	**0.04**	0.31	0.08	0.32	0.65	
Age group	20–39	7.2	888	8956	1299	688	28,963	13,963	5611	3941	26,226	15,078	7111	
	40–49	8.0	714	7663	1014	597	21,518	11,251	4869	3439	23,800	14,049	8262	
	50-	7.9	570	6647	904	542	24,150	12,684	5285	3226	22,688	14,993	6912	
	P-value	0.06	** < 0.01**	** < 0.01**	** < 0.01**	0.10	0.34	0.79	0.93	0.28	0.63	0.98	0.78	
Body mass index	< 18.5	7.9	726	9287	1247	634	23,257	11,324	5276	3850	20,129	10,818	16,097	
	18.5–25	7.5	710	7579	1062	596	23,866	12,513	5149	3235	22,469	14,403	6977	
	> 25	8.5	801	7703	1024	576	29,213	13,762	5457	4480	34,024	18,518	9392	
	P-value	0.13	0.88	0.08	**0.02**	0.68	0.39	0.49	0.92	0.43	**0.03**	0.14	0.27	
Smoking status	No	7.7	727	7874	1095	620	24,308	12,690	5345	2306	16,646	13,580	5122	
	Yes	7.1	712	6256	720	381	23,019	8972	3462	3506	24,294	14,828	7460	
	P-value	0.90	0.99	0.14	** < 0.01**	** < 0.01**	0.49	0.11	0.09	0.66	0.49	0.69	0.93	
Drinking status	No	7.4	715	8072	1111	596	24,363	12,468	5352	3122	21,444	13,679	8157	
	Yes	7.9	734	7634	1056	606	24,064	12,350	5029	1082	12,889	10,270	NA	
	P-value	0.44	0.93	0.20	0.30	0.96	0.59	0.75	0.57	0.50	0.14	0.39	0.47	
Use of immunosuppressive agents	Yes	7.36	386	5462	714	326	18,663	11,195	4255	2435	21,213	13,619	2890	
	No	7.67	736	7859	1086	613	24,432	12,455	5234	3533	24,007	14,778	7698	
	P-value	0.97	0.21	0.16	0.29	**0.04**	0.38	0.65	0.50	0.18	0.33	0.62	0.25	
Hypertension	Yes	7.69	738	7960	1105	343	22,952	12,099	4456	3461	28,089	13,447	3305	
	No	7.49	631	6545	862	624	24,313	12,434	5254	3443	23,314	14,864	8108	
	P-value	0.83	0.19	**0.04**	**0.02**	** < 0.01**	0.82	0.98	0.64	**0.04**	0.80	0.81	0.15	
Diabetes	Yes	6.91	714	6782	968	474	36,522	20,604	8703	4721	41,206	22,461	NA	
	No	7.68	727	7826	1080	602	24,096	12,332	5165	3433	23,659	14,627	7473	
	P-value	0.90	0.62	0.58	0.59	0.46	0.51	0.35	0.34	NA	NA	0.46	NA	
Dyslipidemia	Yes	7.41	648	6398	860	435	20,835	11,324	4732	4721	41,206	22,461	NA	
	No	7.69	735	7955	1101	615	24,500	12,499	5236	3433	23,659	14,627	7473	
	P-value	0.38	0.37	0.13	**0.046**	0.12	0.49	0.72	0.82	0.59	0.29	0.90	NA	
Pre-vaccination antibody titer (AU/mL)	< 25th percentile	–	219	4470	658	264	14,799	6596	2779	1150	13,528	7530	3580	
	25th-75th percentile	–	761	7798	1103	623	26,047	13,553	5402	3454	22,425	14,246	7271	
	> 75th percentile	–	2198	13,649	1685	1271	34,228	19,550	9001	10,267	47,348	30,575	17,348	
	P-value	–	–	** < 0.01**	** < 0.01**	–	** < 0.01**	** < 0.01**	** < 0.01**	–	** < 0.01**	** < 0.01**	** < 0.01**	

		Fever after the first dose		Fever after the second dose			Fever after the third dose				Fever after the fourth dose			
The level of post-vaccination fever	< 37 °C	7.7	722^a^	617^b^	6141	924	536	21,598	11,551	4845	2857	20,482	11,849	5304
	37.0–37.9 °C	7.1	740^a^	752^b^	8674	1168	631	24,479	12,400	5113	3466	23,386	15,195	7988
	≥ 38 °C	6.8	1405	985^b^	10,649	1276	689	31,107	14,839	6480	4955	33,300	21,195	12,558
	P-value	–	0.42	–	** < 0.01**	** < 0.01**	–	**0.01**	0.11	0.15	–	**0.02**	** < 0.01**	**0.03**

		The first and second doses				The second and third doses				The third and fourth doses				
The interval between the doses (days)	< 25th percentile	–	–	–	–	754	27,652	11,701	4669	3910	23,576	12,481	8484	
	25th-75th percentile	–	–	–	–	567	22,615	11,863	5039	3544	22,464	14,207	8120	
	> 75th percentile	–	–	–	–	567	25,439	14,548	6147	2867	27,075	18,550	5844	
	P-value	–	–	–	–	0.06	0.73	0.16	0.20	0.38	0.40	**0.04**	0.28	

P-values were determined using the Wilcoxon rank-sum test or the Jonckheere-Terpstra trend test as applicable.

Significant values are in bold.

^a^Geometric mean antibody titer at S2-0 stratified by fever level after the first dose.

^b^Geometric mean antibody titer at S2-0 stratified by fever level after the second dose.

Significant differences were observed in the GMT ratios (Table 4) based on vaccine product (V2-0/V1-0, V2-4W/V2-0, V2-6M/V2-0), gender (V2-4W/V2-0, V3-3M/V3-0, V3-6M/V3-0), age group (V2-0/V1-0), BMI category (V2-4W/V2-0, V2-6M/V2-0), smoking status (V2-6M/V2-0, V3-2W/V3-0), hypertension (V3-2W/V3-0, V3-3M/V3-0), pre-vaccination antibody titer (V2-4W/V2-0, V2-6M/V2-0, V3-2W/V3-0, V3-3M/V3-0, V3-6M/V3-0, V4-2W/V4-0, V4-3M/V4-0, V4-6M/V4-0), and the interval since the last vaccination (V3-3M/V3-0, V3-6M/V3-0, V4-2W/V4-0, V4-3M/V4-0, V4-6M/V4-0). No significant differences were observed in comparisons based on drinking status, use of immunosuppressive agents, diabetes, dyslipidemia, or post-vaccination fever level at any point.

**Table 4:** Geometric mean fold increases of anti-RBD antibodies and comparison between each category.

		The 1st dose	The second dose		The third dose			The fourth dose		
		V2-0/V1-0	V2-4W/V2-0	V2-6M/V2-0	V3-2W/V3-0	V3-3M/V3-0	V3-6M/V3-0	V4-2W/V4-0	V4-3M/V4-0	V4-6M/V4-0
Administered vaccine product	BNT162b2	91.7	10.9	1.5	40.7	21.1	8.8	6.7	3.7	1.9
	mRNA-1273	304.0	7.1	0.9	33.8	14.3	6.3	7.0	4.6	2.2
	P-value	** < 0.01**	**0.03**	**0.03**	0.40	0.12	0.18	0.80	0.09	0.37
Gender	Male	93.2	9.8	1.5	50.2	30.6	13.3	7.4	4.2	2.5
	Female	95.5	11.2	1.5	39.3	19.7	8.2	6.8	4.3	2.0
	P-value	0.37	**0.04**	0.85	0.11	** < 0.01**	** < 0.01**	0.57	0.72	0.76
Age group	20–39	124.0	10.1	1.5	42.1	20.3	8.2	6.7	3.8	1.7
	40–49	88.8	10.7	1.4	36.0	18.8	8.2	6.9	4.1	2.1
	50-	71.9	11.7	1.6	44.5	23.4	9.7	7.0	4.7	2.6
	P-value	** < 0.01**	0.16	0.73	0.46	0.33	0.30	0.65	0.16	0.14
Body mass index	< 18.5	91.7	12.8	1.7	36.7	17.8	8.3	5.2	2.8	1.4
	18.5–25	95.3	10.7	1.5	40.0	21.0	8.6	7.0	4.5	2.2
	25 <	94.9	9.6	1.3	50.7	23.9	9.5	7.6	4.1	1.8
	P-value	0.93	**0.02**	**0.02**	0.09	0.06	0.42	0.25	0.31	0.62
Smoking status	No	94.5	10.8	1.5	39.2	20.5	8.6	6.9	4.2	2.0
	Yes	101.0	8.8	1.0	60.5	23.6	9.1	7.2	5.9	3.3
	P-value	0.82	0.18	**0.02**	**0.01**	0.44	0.64	0.89	0.35	0.29
Drinking status	No	96.8	11.3	1.6	40.9	20.9	9.0	7.0	4.2	2.1
	Yes	93.4	10.4	1.4	39.7	20.4	8.3	6.9	4.4	2.1
	P-value	0.66	0.16	0.35	0.84	0.82	0.53	0.98	0.75	0.91
Use of immunosuppressive agents	Yes	52.5	14.1	1.85	57.3	34.4	13.1	8.71	5.59	2.22
	No	95.9	10.7	1.48	39.9	20.3	8.54	6.80	4.18	2.10
	P-value	0.19	0.81	0.59	0.17	0.07	0.17	0.22	0.09	0.67
Hypertension	Yes	84.3	10.4	1.37	66.9	35.3	13.0	8.12	3.89	1.86
	No	96.0	10.8	1.50	39.0	19.9	8.42	6.77	4.32	2.13
	P-value	0.34	0.56	0.39	** < 0.01**	**0.02**	0.07	0.33	0.76	0.75
Diabetes	Yes	103	9.50	1.36	77.0	43.4	18.3	8.73	4.76	NA
	No	94.6	10.8	1.49	40.0	20.5	8.57	6.89	4.26	2.10
	P-value	0.83	0.60	0.67	0.14	0.11	0.11	0.51	0.61	NA
Dyslipidemia	Yes	87.5	9.87	1.33	47.9	26.0	10.9	8.73	4.76	NA
	No	95.5	10.8	1.50	39.8	20.3	8.51	6.89	4.26	2.10
	P-value	0.55	0.39	0.24	0.28	0.23	0.31	0.29	0.54	NA
Pre-vaccination antibody titer (AU/mL)	< 25th percentile	–	20.4	3.0	56.1	25.0	10.5	11.8	6.6	3.2
	25th–75th percentile	–	10.2	1.5	41,8	21.8	8.7	6.5	4.1	1.9
	75th percentile <	–	6.2	0.8	26.9	15.4	7.1	4.6	3.0	1.5
	P-value	–	** < 0.01**	** < 0.01**	** < 0.01**	** < 0.01**	** < 0.01**	**0.03**	** < 0.01**	** < 0.01**

		Fever after the first dose	Fever after the second dose		Fever after the third dose			Fever after the fourth dose		
The level of post-vaccination fever	< 37 °C	93.3	10.0	1.5	40.3	21.6	9.0	7.2	4.2	2.0
	37.0–37.9 °C	105.0	11.5	1.6	38.8	19.6	8.1	6.8	4.4	2.2
	≥ 38 °C	207.0	10.8	1.3	45.1	21.5	9.4	6.7	4.3	1.9
	P-value	0.17	0.13	0.71	0.69	0.97	0.87	0.57	0.78	0.89

			The first and second doses		The second and third doses			The third and fourth doses		
The interval between the doses (days)	< 25th percentile	–	–	–	36.7	15.5	6.2	6.0	3.2	1.22
	25th-75th percentile	–	–	–	39.9	20.9	8.9	6.3	4.0	1.83
	75th percentile <	–	–	–	44.9	25.7	10.8	9.5	6.5	3.8
	P-value	–	–	–	0.11	** < 0.01**	** < 0.01**	**0.03**	** < 0.01**	** < 0.01**

Fold increases (V4-6M/V4-0) were unavailable for 56 cases. P-values were calculated using the Wilcoxon rank-sum test or the Jonckheere-Terpstra trend test as appropriate.

Significant values are in bold.

### Multivariate analysis to identify predictors of anti-RBD antibody titers (Table 5)

**Table 5:** Multivariate analysis to identify predictors of anti-RBD antibody titers (AU/mL).

Predicting variable*	After the first dose, N = 467 (multiple regression model)			After the second dose, N = 467 (linear mixed model)			After the third dose, N = 157 (linear mixed model)			After the fourth dose, N = 89 (linear mixed model)		
	Antibody titers at V2-0			Antibody titers at V2-4W, V2-3M, V2-6M			Antibody titers at V3-2W, V3-3M, V3-6M			Antibody titers at V4-2W, V4-3M, V4-6M*		
	β	Std. error	P-value	β	Std. error	P-value	β	Std. error	P-value	β	Std. error	P-value
mRNA-1273 (ref. BNT162b2)	0.397	0.129	** < 0.01**	0.121	0.061	**0.048**	0.133	0.098	0.17	− 0.049	0.044	0.27
Male (ref. female)	− 0.028	0.043	0.52	− 0.001	0.026	0.97	0.158	0.063	**0.01**	0.161	0.064	**0.01**
Age (years, continuous)	− 0.008	0.002	** < 0.01**	− 0.002	0.001	0.06	0.003	0.002	0.17	0.003	0.002	0.17
Body mass index (kg/m^2^, continuous)	0.012	0.006	0.06	− 0.004	0.003	0.19	0.002	0.006	0.69	0.001	0.007	0.85
Current smoker (ref. non-smokers)	0.004	0.097	0.97	− 0.109	0.051	**0.03**	0.017	0.077	0.87	0.058	0.059	0.33
Current drinker (ref. non-smokers)	0.001	0.038	0.98	− 0.018	0.020	0.36	− 0.041	0.037	0.26	− 0.176	0.194	0.37
Use of immunosuppressants	− 0.288	0.134	**0.03**	− 0.032	0.071	0.65	0.041	0.1070	0.70	0.101	0.081	0.21
Hypertension	− 0.021	0.069	0.77	− 0.007	0.037	0.85	0.038	0.081	0.19	− 0.060	0.063	0.34
Diabetes	− 0.110	0.138	0.43	− 0.010	0.073	0.89	0.261	0.1657	0.12	− 0.014	0.1711	0.93
Hyperlipidemia	0.023	0.070	0.74	− 0.034	0.037	0.36	0,001	0.076	0.99	− 0.079	0.0633	0.21
Pre-vaccination antibody titer (AU/mL, continuous)	0.133	0.089	0.13	0.426	0.025	** < 0.01**	0.710	0.075	** < 0.01**	0.578	0.052	** < 0.01**
Post-vaccination fever category	0.030	0.058	0.60	0.047	0.014	** < 0.01**	0.035	0.026	0.19	0.060	0.025	**0.01**
Interval since the last dose (days, continuous)	Not included in the model						0.004	0.001	** < 0.01**	0.000	0.001	** < 0.01**

Antibody titers refer to anti-receptor-binding-domain antibody titers. All antibody titers were base 10 log transformed. The post-vaccination fever category was classified as follows: < 37 °C, 37.0–37.9 °C, > 38 °C. The model included all variables presented in the table and was adjusted for the number of days from vaccination to each blood collection. For linear mixed models, participant ID numbers were included as a random-effects variable.

Significant values are in bold.

*V4-6M antibody titer was missing in 56 cases.

In individuals vaccinated with mRNA-1273 vaccine compared to those with BNT162b2, significant increases in antibody titers were observed after the first (P < 0.01) and second doses (P = 0.047). There was a significant increase in antibody titers after the third and fourth doses in male compared to female (both P = 0.01). Older age was associated with a significant decrease in antibody titers after the first (P < 0.01) and second doses (P = 0.02). There was no significant association between BMI and antibody titers after vaccination. Current smokers showed a significant decrease in antibody titers after the second dose (P = 0.04), whereas no significant association was observed with respect to drinking status. Use of immunosuppressive agents was significantly associated with antibody titers after the first dose (P = 0.03). No significant associations were observed between underlying diseases (hypertension, diabetes, dyslipidemia) and post-vaccination antibody titers. Higher pre-vaccination antibody titers were significantly associated with higher antibody titers after the second, third, and fourth doses (P < 0.01 for all). Higher levels of post-vaccination fever were associated with significant increases in antibody titers after the second (P < 0.01) and fourth doses (P = 0.02). Additionally, longer intervals since the last vaccinations were significantly associated with increases in antibody titers after the third and fourth doses (P < 0.01 for both).

## Discussion

This study examined the kinetics of antibody titers over four doses and found that there was variability in titers after the first vaccination between individuals. However, subsequent doses resulted in less variability, with rapid increases followed by gradual declines over a period of 6 months. Factors associated with higher post-vaccination antibody titers included mRNA-1273 vaccine (first and second dose), male gender (third and fourth doses), younger age (first and second dose), non-smoking status (second dose), non-use of immunosuppressive agents (first dose), higher pre-vaccination antibody titers (second, third, and fourth doses), higher level of post-vaccination fever (second and fourth doses), and longer intervals since the last vaccinations (third and fourth doses).

After the second, third and fourth doses, the antibody titers increased rapidly after vaccination in all participants. Also, despite the similar pre-vaccination antibody titers at the second and the third dose, the post-vaccination antibody titers (GMT and GMTR) were higher after the third dose compared to the second dose. This result is consistent with other studies and supports the notion that the third dose has superior immunogenicity compared to the second dose^26^. On the other hand, although the GMT of the pre-vaccination antibody titer was clearly higher before the fourth dose than the third dose, the GMT of post-vaccination antibody titer were similar values. This can be attributed to the fact that, as described in other studies^18,19,27^, antibody titers do not rise further once they reach peak.

Compared with BNT162b2 vaccine recipients, mRNA-1273 vaccine recipients showed higher antibody levels after the first and second doses, but no significant difference was observed after the third and fourth doses. This difference is likely since the amount of active ingredient included in each dose of mRNA-1273 was higher for the first and second doses (0.10 mg in 0.5 ml) compared to BNT162b2 (0.0375 mg in 0.3 ml). However, for the third and fourth doses of mRNA-1273, the amount was reduced (0.05 mg in 0.25 ml) and became closer to that of the BNT162b2 vaccine^19^.

Regarding gender, some previous studies reported that the antibody titer after the second vaccination was higher in females^7,10,15^. In contrast, in the present study, antibody titers in male significantly increased after the third and fourth vaccinations. Similar results have been reported in a previous study^15^, but the mechanism is not yet clear and requires further verification.

Similar to our finding, several studies of first and second doses have shown that older age is significantly associated with lower antibody levels after vaccination^7–11,13,15^. One possible explanation is immunosenescence, the aging of the immune system, which may delay the production of specific antibodies against new antigens in older individuals^28^. However, it has been reported that the effect of age diminishes after the third vaccination^15,29–31^, which is also consistent with the results of this study. After the third vaccination, it has been demonstrated that there are sufficient virus-specific memory B cells even in the elderly, leading to rapid antibody production^29^.

The present study found that BMI had a little effect on increasing antibody levels. However, other studies have reported that obese influence the decline in antibody titers after the second vaccination in Japanese population^12,15^. These results need to be validated in other populations that include a more diverse range of underweight and obese individuals.

Current smokers had significantly lower antibody levels compared to nonsmokers after the second dose. This decline was particularly evident in antibody levels 6 months after vaccination, suggesting a potential negative impact of smoking on long-term antibody responses. Similar results have been reported in previous studies^10,12,15^. Furthermore, tobacco smoke components, mainly nicotine, have been shown to reduce immune responses^32^. However, no significant difference in antibody levels was observed after the third and fourth doses in the present study. The low prevalence of smokers and the small number of subjects for the third and fourth dose analysis may have reduced the statistical power.

The present study found no significant effect of alcohol drinking status on antibody titers. However, previous studies have reported that daily alcohol consumption reduces antibody titers after the second^13,15^ and third doses^17^. The results may have been inconsistent because the present study did not consider the frequency or amount of alcohol consumption.

Previous research has reported a significant decrease in antibody levels after the first, second, and third doses of vaccination in individuals using immunosuppressive agents^15^. Although the proportion of immunosuppressants users among participants in this study was small, leading to no significant decrease after the second, third, and fourth doses of vaccination, use of immunosuppressants was considered to have a significant impact, particularly on the antibody levels after the first dose of vaccination. On the other hand, the presence of hypertension, diabetes, and dyslipidemia was considered to have no significant impact on antibody levels after vaccination.

Higher pre-vaccination antibody levels before the second, third, and fourth doses were significantly associated with higher post-vaccination antibody levels both in comparing GMTs and in multivariate analysis using linear mixed models. In contrast, when examining relationships with GMT ratios, individuals with lower pre-vaccination antibody titers had significantly higher GMT ratios after vaccination. This is consistent with observations in previous studies comparing antibody levels after vaccination^27,33^, suggesting a caution when interpreting GMT ratios in antibody levels.

Previous studies have found a positive association between fever levels and antibody titers after the second dose^14^ and no association between fever levels and GMT ratios after the third dose^34^, which is consistent with our findings. In the present study, even in multivariate analysis adjusted for pre-vaccination antibody titers, fever levels after the second and fourth doses were significantly associated with post-vaccination antibody titers. However, as shown in Supplementary Table 1, it is noteworthy that individuals with high antibody titers before the second dose had significantly higher levels of fever after vaccination. There remains a discussion on whether high fever after vaccination leads to high antibody titers or if high pre-vaccination antibody titers cause high fever after vaccination.

In the present study, we found that longer intervals between the second and third doses and between the third and fourth doses were significantly associated with higher antibody titers even after adjusting for pre-vaccination antibody levels. A potential mechanism includes the attenuation of the immune response when the same vaccine is administered multiple times over a short period of time^35,36^. The impact of the interval between the first and second doses could not be evaluated in the present study as most participants were vaccinated according to the interval described in the package insert. However, previous research has reported that longer intervals between the first and second doses lead to increased antibody levels^9,37,38^, suggesting that longer dosing intervals of COVID-19 mRNA vaccines may result in improved immune response.

Compared to our previous article^7^, this study delves deeper into the factors impacting antibody levels following the primary vaccination series (first and second doses), including age as previously mentioned. Additionally, it includes insights on vaccine products, the use of immunosuppressants, and post-vaccination fever, which were identified as critical factors affecting antibody levels post-vaccination. Age, vaccine products, and use of immunosuppressive agents significantly affect antibody titers after the first dose, while smoking, pre-vaccination antibody levels, and post-vaccination fever significantly influence titers after the second dose. However, for subsequent doses (third and fourth), unlike the primary series, factors such as age, immunosuppressive therapy, and smoking do not significantly impact post-vaccination antibody levels. Booster dose administration is especially crucial for individuals prone to a decline in antibody titers after the second dose, notably the elderly, those using immunosuppressive agents, and smokers. Administering additional doses with short intervals for the third and fourth doses may reduce effectiveness compared to spacing out vaccination intervals. However, individuals with longer intervals between vaccinations, such as over a year, were not included in this study, so the optimal vaccination interval remains undetermined. Further research is necessary to establish appropriate vaccination intervals.

The strength of this study lies in its extensive evaluation of the immunogenicity of monovalent mRNA vaccines targeting the wild-type SARS-CoV-2, spanning from the pre-first to the post-fourth doses, conducted through a continuous prospective cohort study. The study meticulously analyzed factors influencing the rise in antibodies following the first, second, third, and fourth vaccine doses, encompassing variables like smoking, post-vaccination fever, and dose intervals, while accounting for pre-vaccination antibody titers. Additionally, a notable strength was the ability to observe changes in immunogenicity solely attributable to vaccination in a population naïve to SARS-CoV-2.

However, this study's limitations include a relatively small cohort for assessing immunogenicity after the third and fourth doses. In particular, the number of participants who received the fourth dose without a history of SARS-CoV-2 infection was limited, with a considerable number of individuals receiving their next dose prior to the six-month mark. Therefore, the analysis of antibody titers after the fourth dose was constrained, potentially affecting statistical power, and necessitating careful interpretation of the results. Additionally, only anti-RBD antibody titers were used as an immunogenicity indicator, neglecting neutralizing antibody titers and cell-mediated immunity, despite their significant roles in vaccine response.

## Conclusion

In this study, we continuously evaluated the kinetics of antibody titers after the first four doses in SARS-CoV-2-naïve individuals. The effect of age on antibody titer increase was significant after the first and second doses, but not significant after the third and fourth doses. Smoking was found to have a potentially negative impact on antibody titer increase, while higher post-vaccination fever had a positive impact on antibody titer increase. Longer intervals between doses may result in more efficient vaccine immunogenicity. The results of this study are expected to contribute to a better understanding of the immune response to mRNA vaccines and aid in the development of individualized vaccination schedule based on personal characteristics. Determining an optimal vaccination strategy requires further evaluation in larger populations and the use of other measures, including neutralizing antibodies and cell-mediated immunity.

### Supplementary Information

Supplementary Information.

## Data Availability

The data supporting the findings of this study are available from the Graduate School of Medicine, Osaka Metropolitan University, but access to these data is restricted as they were used under license for the current study and are not publicly available. However, the data can be obtained from the corresponding author upon reasonable request and with the permission of the Graduate School of Medicine, Osaka Metropolitan University.
