# Supplementary material for: Factors impacting antibody kinetics, including fever and vaccination intervals, in SARS-CoV-2-naïve adults receiving the first four mRNA COVID-19 vaccine doses
Source: Sci Rep. 2024 Mar 27;14:7217. doi: 10.1038/s41598-024-57931-0 (PMC10973463; doi:10.1038/s41598-024-57931-0)
Supplement: Supplementary file 1 — Supplementary Information. [file 41598_2024_57931_MOESM1_ESM.docx]

**Supplementary figure 1.**


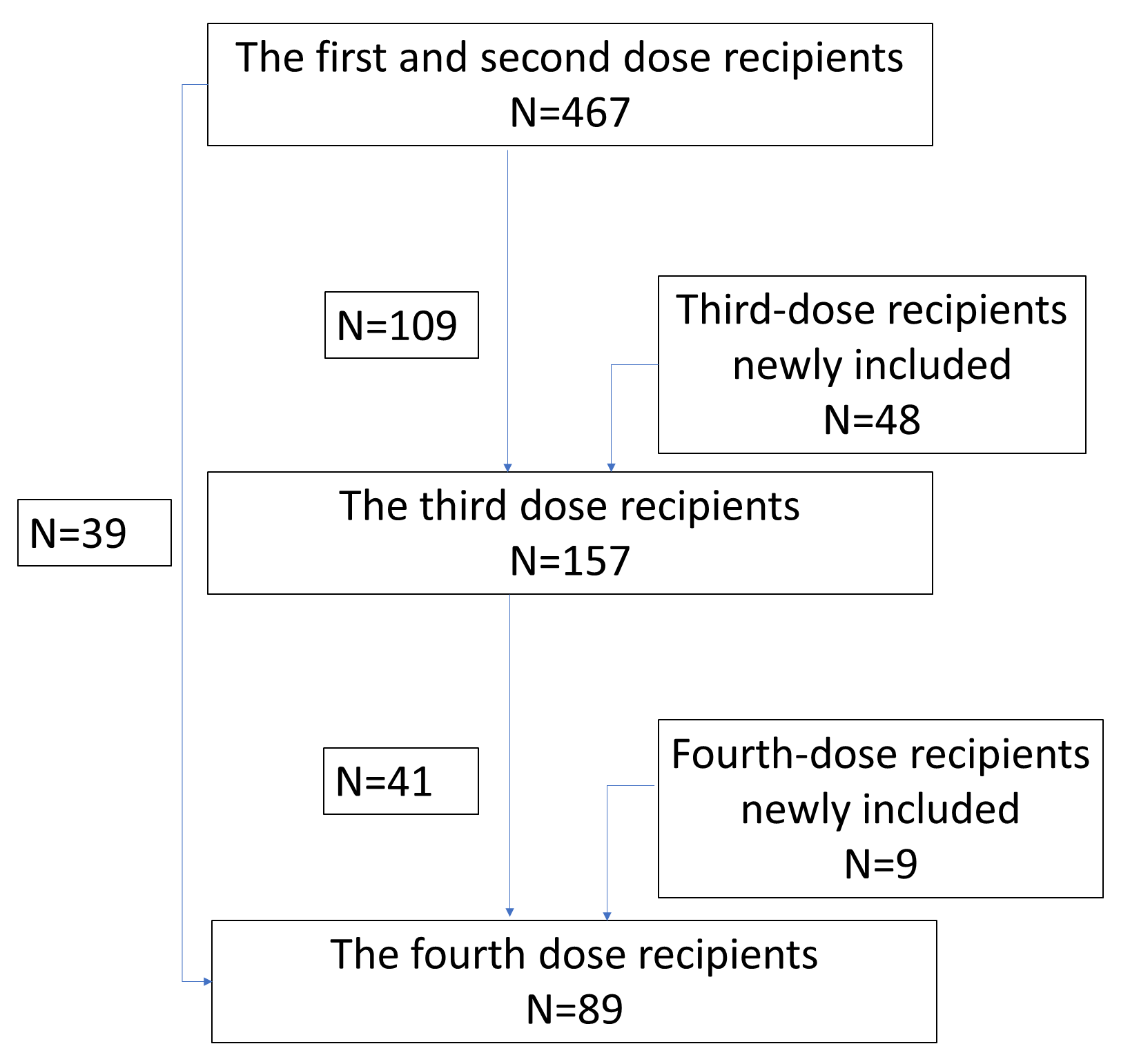


Flow diagram of who is included in the analysis for each vaccination.

A total of 27 participants consecutively included in the analysis of the first, second, third, and fourth doses.

**Supplementary table 1.**

|  | | *Pre-vaccination antibody titer category, n (%)*  *(1:* <25th percentile, 2: 25th-75th percentile, 3: >75th percentile) | | | | | | | | | | | |
| --- | --- | --- | --- | --- | --- | --- | --- | --- | --- | --- | --- | --- | --- |
|  |  | *The first dose*  *(V0-1, N=467)* | | | *The second dose*  *(V2-0, N=467)* | | | *The third dose*  *(V3-0, N=157)* | | | *The fourth dose*  *(V4-0, N=89)* | | |
|  | | *1* | *2* | *3* | *1* | *2* | *3* | *1* | *2* | *3* | *1* | *2* | *3* |
| *The level of post-vaccination fever* | *<37℃* | ― | *370*  *(90)* | *53 (98)* | 58 (50) | *99*  *(43)* | *34*  *(29)* | *17*  *(44)* | *31*  *(39)* | *13*  *(33)* | *12 (55)* | *20 (44)* | *4*  *(18)* |
|  | *37.0-37.9℃* | ― | *40*  *(10)* | *1 (2)* | 47 (40) | *99*  *(43)* | *49*  *(42)* | *16*  *(41)* | *37*  *(47)* | *18*  *(46)* | *6*  *(27)* | *19 (42)* | *10 (46)* |
|  | *≥38℃* | ― | *3 (1)* | *0 (0)* | 12 (10) | *35*  *(15)* | *34*  *(29)* | *6*  *(15)* | *11*  *(14)* | *8*  *(21)* | *4*  *(18)* | *6*  *(13)* | *8*  *(36)* |
|  | *P-value** | *0.12* | | | ***<0.01*** | | | *0.83* | | | *0.06* | | |

Association between pre-vaccination antibody titer (AU/mL) and the level of post-vaccination fever.

*P-values were tested using the chi-square test or Fisher's exact test as appropriate.
